# A Rare Case of Profound Pulmonary Venous Malformation in an Elderly Individual With Three Major Comorbidities: Genetic Insights

**DOI:** 10.7759/cureus.94471

**Published:** 2025-10-13

**Authors:** Andrey Frolov, Sophia Izhar, Samantha M Spence, Beker Karadaghy, Madeleine Schwab, Yun Tan, Daniel T Daly

**Affiliations:** 1 Department of Surgery, Center for Anatomical Science and Education, Saint Louis University School of Medicine, St. Louis, USA

**Keywords:** accessory pulmonary veins, angiogenesis, cardiogenesis, genetics, pulmonary venous development

## Abstract

Pulmonary veins play a very important role in normal human physiology by providing oxygenated blood flow to the heart, as well as in human pathophysiology by inducing and controlling atrial fibrillation and participating in the propagation of pulmonary neoplasms, such as non-small cell lung cancer. Gaining additional insights into the mechanism(s) of pulmonary venous development (PVD) would benefit our understanding of heart and lung function under normal and clinical conditions. In the current report, we present the results of postmortem genetic screening of an 87-year-old male with a remarkably high number of pulmonary veins - 12 in total - with normal connection, who died of arteriosclerotic heart disease and heart failure with additional comorbidities being prostate and colon cancers. To advance our understanding of molecular mechanisms underlying such anomalous PVD, genetic screening was performed by whole exome sequencing (WES) using the Illumina next-generation sequencing (NGS) platform. The screening revealed 80 genes with rare (minor allele frequency, MAF ≤ 0.01) pathological/deleterious variants. The functional annotation of the affected genes allowed their grouping into subcategories, including angiogenesis, cardiogenesis, cardiovascular disease, heart failure, prostate cancer, and colon cancer. The respective results were consistent with the polygenic nature of the present case, with a significant pleiotropic component and pointed toward an important role of the CRACD gene in the observed anomalous PVD. The other important finding in our study was the apparent existence of a heart failure↔colorectal/prostate cancer axis with a potential involvement of the pleiotropic C2ORF88 gene. The latter, by virtue of its involvement in cardiogenesis and, hence, angiogenesis, could also serve as a hub gene in the putative angiogenesis↔heart failure↔cancer network, which merits further investigation using a large clinical database(s).

## Introduction

Pulmonary veins represent a unique anatomical structure because they are the only type of veins in the human body that carry oxygenated blood. The main physiological function of pulmonary veins is to provide oxygenated blood flow from both lungs to the heart [1], with an additional important function being regulation of pulmonary circulation via active vasomotion [2]. Pulmonary venous development (PVD) is a complex process that is intricately linked to cardiogenesis, the prenatal development of the heart, and commences at 27-29 days of gestation [3,4]. Normal PVD yields four pulmonary veins that drain into the left atrium, and as such, this pattern is present in 60-70% of the population [5], whereas approximately 38% of the population shows different anatomical configurations [6]. The pulmonary vein pathophysiology is mostly associated with atrial fibrillation [1], non-small cell lung cancer propagation [1], and pulmonary venous stenosis [1,3]. The latter is becoming more common as a result of more aggressive treatment of atrial fibrillation by radiofrequency ablation [1,3]. Despite the very important physiological role of pulmonary veins, there is a paucity of information regarding the molecular mechanism(s) governing their development, particularly the PVD genetic underpinnings. The information with regards to PVD genetic foundation is less scarce in the case of the total anomalous pulmonary venous connection (TAPVC), where a number of candidate genes have been identified, pointing toward TAPVC's polygenic nature [7,8]. Therefore, gaining new insights into the PVD genetic underpinnings would be highly beneficial for our understanding of such complex developmental processes and its associated pathophysiology. This task was approached in the current report by using the next-generation sequencing (NGS) platform for the whole exome sequencing (WES) genetic screening of DNA extracted postmortem from the body of an elderly individual with a remarkably high number of pulmonary veins (12 in total) with normal connections. The respective in-depth anatomical characterization was presented in our recent publication [9]. Because this individual had three major comorbidities - arteriosclerotic heart disease as well as prostate and colon cancers - the respective studies allowed not only identification of CRACD as a potential PVD gene but also pointed toward pleiotropic C2ORF88 as a hub gene in the putative angiogenesis ↔ heart failure ↔ cancer network.

## Case presentation

Body donor

The body of an 87-year-old male was received through the Saint Louis University (SLU) Gift Body Program with signed informed consent from the donor. The self-reported medical history included colon cancer surgery, heart bypass surgery, and prostate cancer radiation. The donor’s causes of death were congestive heart failure and arteriosclerotic heart disease.

Genetic analysis

The postmortem genetic screening by WES on the Illumina NGS platform (San Diego, CA, USA) and the respective bioinformatics analysis were performed exactly as previously described [10]. The cumulative exome coverage for 50x depth of coverage was 94%.

Results

**Figure 1:**
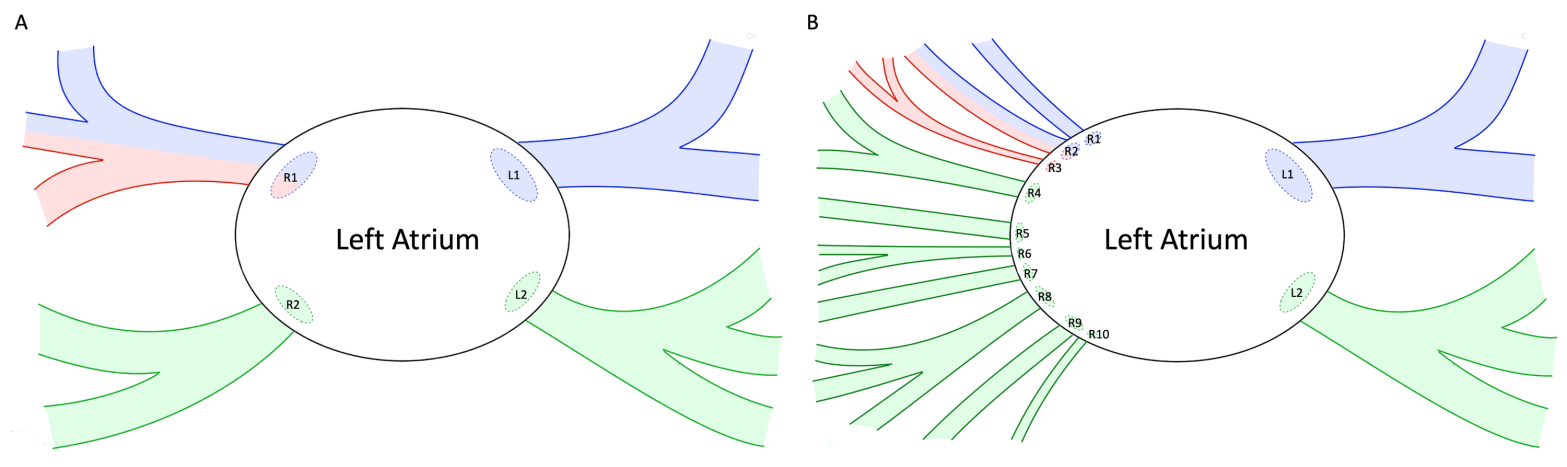
Aberrant pulmonary venous anatomy observed in the present case. A: Typical pulmonary vein (PV) anatomy. B: Aberrant PV anatomy observed in the present case. PVs labeled “R” drain the right lung, and PVs labeled “L” drain the left lung. PVs outlined in blue represent those draining the upper lobe. PVs outlined in red represent those draining the middle lobe. PVs outlined in green represent those draining the lower lobe. R1 (A), representing the right superior pulmonary vein, is shaded in both blue and red to illustrate its physiologic drainage of both the upper and middle lobes of the right lung. Similarly, R2 (B) was found to drain both the upper and middle lobes of the right lung and is depicted accordingly. This diagram (B) is based on our recent publication describing the anatomy of this case [9]. Image credit: The authors

The anatomical examination revealed anomalous PVD in the donor, resulting in the presence of significantly more pulmonary veins than anticipated. Expected pulmonary venous anatomy dictates the presence of four pulmonary veins connecting to the left atrium, with two on the left and two on the right side. In this case, the donor had the expected two left-sided pulmonary veins, but 10 right-sided pulmonary veins (Figure 1). Literature consistently reports variations up to a total of six pulmonary veins; however, there is no documented case in the English language with 12 or more pulmonary veins. Notably, the right-sided vessels had a total cross-sectional area that was smaller than expected. This reduced cross-sectional area and subsequently increased vascular resistance may contribute to the development of pulmonary hypertension. The respective results were presented in further detail in our recent publication [9]. By investigating the underlying anatomic variations in the context of his underlying medical conditions (heart disease, prostate cancer, and colon cancer) and normal embryologic development, the genetic underpinnings can begin to be elucidated, so that clinical management may be guided. The genetic screening of the donor by WES revealed 80 rare (minor allele frequency, MAF ≤ 0.01) pathological/deleterious variants (Table 1).

**Table 1:** Complete list of genes with rare pathological/deleterious variants in the present case. All genes with rare (minor allele frequency, MAF ≤ 0.01) pathological/deleterious variants (n=80) were identified by whole exome sequencing (WES) using the Illumina next-generation sequencing (NGS) platform. The gene-to-protein name conversion was performed using the GeneCards database. Gene names are organized in alphabetical order.

Gene	Protein Function
ABCB9	ATP Binding Cassette Subfamily B Member 9.
ANTXR1	ANTXR Cell Adhesion Molecule 1 (TEM8).
ARMT1	Acidic Residue Methyltransferase 1.
ASB10	Ankyrin Repeat And SOCS Box Containing 10.
ATM	ATM Serine/Threonine Kinase.
BDP1	B Double Prime 1, Subunit of RNA Polymerase III.
C2ORF88	Chromosome 2 Open Reading Frame 88 (Small Membrane A-Kinase Anchor Protein, SMAKA Protein).
CD19	CD19 Molecule.
CIBAR2	CBY1 Interacting BAR Domain Containing 2 (FAM92B).
CITED4	Cbp/P300 Interacting Transactivator With Glu/Asp Rich Carboxy-Terminal Domain 4.
CMA1	Chymase 1.
COL22A1	Collagen Type XXII Alpha 1 Chain.
CPXM2	Carboxypeptidase X, M14 Family Member 2.
CRACD	Capping Protein Inhibiting Regulator of Actin Dynamics.
CTSZ	Cathepsin Z.
CYB5D2	Cytochrome B5 Domain Containing 2.
DNASE1	Deoxyribonuclease 1.
EFL1	Elongation Factor Like GTPase 1.
ESYT3	Extended Synaptotagmin 3.
ETFDH	Electron-Transferring-Flavoprotein Dehydrogenase.
FAT1	FAT Atypical Cadherin 1.
FBLN2	Fibulin 2.
FBXO36	F-Box Only Protein 36.
FHDC1	FH2 Domain Containing 1 (INF1).
FLACC1	Flagellum-Associated Containing Coiled-Coil Domains 1.
FOS	Fos Proto-Oncogene, AP-1 Transcription Factor Subunit.
FOXD4L1	Forkhead Box D4 Like 1.
GAPDHS	Glyceraldehyde-3-Phosphate Dehydrogenase, Spermatogenic.
GNL1	G Protein Nucleolar 1 (Putative).
GREB1	Growth Regulating Estrogen Receptor Binding 1.
ITGB3	Integrin Subunit Beta 3.
KIF6	Kinesin Family Member 6.
KIR2DL1;KIR2DL2;KIR2DL3;KIR2DS2;KIR2DS3;LOC102725023	Killer Cell Immunoglobulin-Like Receptor, Two Ig Domains And Long Cytoplasmic Tail 1.
KRT12	Keratin 12.
KRT38	Keratin 38.
KRT6B	Keratin 6B.
KRT82	Keratin 82.
LDHAL6B	Lactate Dehydrogenase A Like 6B.
METRN	Meteorin, Glial Cell Differentiation Regulator.
MIER2	MIER Family Member 2.
MRI1	Methylthioribose-1-Phosphate Isomerase 1.
MUC21	Mucin 21, Cell Surface Associated.
MXRA5	Matrix Remodeling Associated 5.
MYO5B	Myosin VB.
NAV2	Neuron Navigator 2.
NDUFA10	NADH:Ubiquinone Oxidoreductase Subunit A10.
NINL	Ninein Like.
NUAK1	NUAK Family Kinase 1.
NUDT19	Nudix Hydrolase 19.
OR1L6	Olfactory Receptor Family 1 Subfamily L Member 6.
OR5H2	Olfactory Receptor Family 5 Subfamily H Member 2.
PKD2L2	Polycystin 2 Like 2, Transient Receptor Potential Cation Channel.
PNMA8A	PNMA Family Member 8A.
PRAMEF6	PRAME Family Member 6.
PRMT9	Protein Arginine Methyltransferase 9.
RGL1	Ral Guanine Nucleotide Dissociation Stimulator Like 1.
RIOK3	RIO Kinase 3.
RNF207	Ring Finger Protein 207.
RRP12	Ribosomal RNA Processing 12 Homolog.
SERPINB2	Serpin Family B Member 2 (PAI2).
SFXN5	Sideroflexin 5.
SHCBP1	SHC Binding And Spindle Associated 1.
SIDT1	SID1 Transmembrane Family Member 1.
SIGLEC6	Sialic Acid Binding Ig Like Lectin 6.
SIPA1L3	Signal Induced Proliferation Associated 1 Like 3.
SPATA20	Spermatogenesis Associated 20.
TMEM253	Transmembrane Protein 253.
TMPRSS5	Transmembrane Serine Protease 5.
TRIM35	Tripartite Motif Containing 35.
TRPM1	Transient Receptor Potential Cation Channel Subfamily M.
TRPM4	Transient Receptor Potential Cation Channel Subfamily M.
TTC38	Tetratricopeptide Repeat Domain 38.
TTN	Titin.
TVP23C	Trans-Golgi Network Vesicle Protein 23 Homolog C.
TYR	Tyrosinase.
UCKL1	Uridine-Cytidine Kinase 1 Like 1.
XDH	Xanthine Dehydrogenase.
ZBTB24	Zinc Finger And BTB Domain Containing 24.
ZFP2	ZFP2 Zinc Finger Protein.

Taking into account the intrinsic link between cardiogenesis and the development of new blood vessels, angiogenesis [3], as well as the presence of coexisting medical conditions in the donor such as arteriosclerotic heart disease (eventually leading to heart failure), colon and prostate cancers (see above), the genetic variants most relevant to the present case were grouped into the following categories: Angiogenesis; Cardiogenesis; Angiogenesis and Cardiogenesis; Cardiovascular Disease; Heart Failure; Cancer; Heart Failure and Cancer. The respective data are presented in Table 2.

**Table 2:** Selected genes with rare pathological/deleterious variants associated with the present case. Selected genes with rare (minor allele frequency, MAF ≤ 0.01) pathological/deleterious variants organized into groupings - Angiogenesis, Cardiogenesis, Cardiogenesis and Angiogenesis, Cardiovascular Disease, Heart Failure, Cancer, and Heart Failure and Cancer - based on their established or suspected involvement in these disease processes. Within each group, genes are further identified based on their correlations with the comorbidities present in this case - colorectal and prostate cancer. * Colorectal cancer; ** Prostate cancer; ***Colorectal cancer and prostate cancer.

Biological Process/Pathological Condition	Gene Name
Angiogenesis	CMA1, CRACD, EFL1, FBXO36, ITGB3, METRN, NDUFA10, SERPINB2
Cardiogenesis	C2ORF88, CIBAR2, FOXD4L1, GNL1, MRI1, NUAK1
Cardiogenesis and Angiogenesis	ANTRX1, FBLN2, FOS, MIER2, MYO5B, TRPM4, ZFP2
Cardiovascular Disease	C2ORF88, COL22A1, CTSZ, ESYT3, FOS, GREB1, KIF6, MXRA5, RNF207, RRP12, SFXN5, SHCBP1, SIGLEC6, TMPRSS5, TTC38, TVP23C
Heart Failure	ARMT1, CPXM2, SERPINB2
Cancer	ANTRX1*, C2ORF88***, COL22A1, CRACD*, CTSZ, CYB5D2***, ESYT3, ETFDH, FAT1, FBLN2, FHDC1, FOXD4L1, GREB1*, ITGB3, MUC21, MYOB5, NINL, NUAK1, RIOK3, RNF207, RRP12*, SHCBP1**, SIDT1, SIGLEC6*, SIPA1L3, SPATA20*, TMEM253, TMPRSS5**, TRPM4***, TTC38*, TVP23C, TYR, XDH
Heart Failure and Cancer	ABCB9, ATM, CD19, CITED4, MXRA5*, TRIM35, TRPM1**, TTN***, UCKL1**

## Discussion

The results of genetic screening were consistent with a polygenic nature of aberrant PVD in the present case, meaning this condition is likely caused by a combination of multiple genetic variants. Indeed, there were multiple entries in the Angiogenesis as well as Cardiogenesis groups (Table 2) signifying an interlink between these two major developmental processes [3]. In the Angiogenesis group, there was a notable presence of the CRACD gene with the only biallelic variant (NM_020722:exon8:c.A1033G:p.R345G) in the entire dataset. CRACD, also known as CRAD, is involved in the negative regulation of barbed-end actin filament capping [11]. Because actin capping plays a key role in the regulation of cell motility [12,13] and angiogenesis [14,15], one could suggest an important role for CRACD in the observed aberrant PVD. The other interesting gene, C2ORF88, was present in three groups: Cardiogenesis, Cardiovascular Disease, and Cancer. This gene was differentially regulated in the bicuspid aortic valve with leaflet redundancy [16] as well as in colon cancer [17]. Yet, C2ORF88 was also related to atrial fibrillation [18] and prostate cancer [19]. Since atrial fibrillation could lead to heart failure [20], C2ORF88 could serve as an important player in the heart failure ↔ colorectal/prostate cancer axis, apparently present in the current case. Intriguingly, there was also a number of genes linked to both angiogenesis and cardiogenesis (Table 2), which could point to a much closer link between these two biological processes, potentially regulated by pleiotropic genes.

The data obtained in the current study could also provide novel insights into the molecular basis of an important link between heart failure and cancer [21,22] due to common risk factors for these pathological conditions [23]. The genetic screening of the donor who had colon cancer, prostate cancer, and succumbed to heart failure identified three genes linked to the latter - ARMT1, CPXM2, and SERPINB2 - along with a plethora of genes associated with cancer, including those of colorectal and prostate (Table 2). Interestingly, in addition to genes linked to colorectal or prostate cancer, there were also two genes known to be associated with both of those cancer types: CYB5D2 [24,25] and TRPM4 [26,27]. More interestingly, there was also a group of pleiotropic genes - ABCB9, ATM, CD19, CITED4, MXRA5, TRIM35, TRPM1, TTN, UCKL1 - linked to both heart failure and cancer. Within these pleiotropic genes, MXR5 [28,29] was associated with colorectal cancer [30]; TRPM1 [31] and UCKL1 [32,33] were linked to prostate cancer [34,35], and TTN [36] was associated with both cancer types [37].

Despite providing important insights into the putative mechanism(s) underlining a pulmonary venous malformation, the current case involved one individual who was studied postmortem. Therefore, the current report can only be viewed as an initial step for the antemortem studies in a clinical setting with a large cohort of patients having similar malformations and/or using respective clinical database(s).

## Conclusions

The profound pulmonary venous malformation observed in the donor had most likely pleiotropic genetic underpinnings with a potential involvement of an aberrant barbed-end actin filament capping due to the rare biallelic mutation in the CRACD gene. The pleiotropic C2ORF88 gene could possibly serve as a hub gene linking aberrant angiogenesis, heart failure, and cancer, all present in the donor. These results warrant a study of similar cases using a large clinical database(s).

## References

[1] Porres DV, Morenza OP, Pallisa E, Roque A, Andreu J, Martínez M (2013). Learning from the pulmonary veins. Radiographics.

[2] Gao Y, Raj JU (2005). Role of veins in regulation of pulmonary circulation. Am J Physiol Lung Cell Mol Physiol.

[3] Latson LA, Prieto LR (2007). Congenital and acquired pulmonary vein stenosis. Circulation.

[4] van den Berg G, Moorman AF (2011). Development of the pulmonary vein and the systemic venous sinus: an interactive 3D overview. PLoS One.

[5] Lacomis JM, Goitein O, Deible C, Schwartzman D (2007). CT of the pulmonary veins. J Thorac Imaging.

[6] Kato R, Lickfett L, Meininger G (2003). Pulmonary vein anatomy in patients undergoing catheter ablation of atrial fibrillation: lessons learned by use of magnetic resonance imaging. Circulation.

[7] Shi X, Huang T, Wang J (2018). Next-generation sequencing identifies novel genes with rare variants in total anomalous pulmonary venous connection. EBioMedicine.

[8] Shi X, Lu Y, Sun K (2020). Research progress in pathogenesis of total anomalous pulmonary venous connection. Methods Mol Biol.

[9] Schwab M, Spence S, Izhar S, Frolov A, Tan Y, Daly DT (2025). Anatomical insights into profound pulmonary venous malformation in an elderly individual: a report of a rare cadaveric case. Cureus.

[10] Frolov A, Guzman MA, Hayat G, Martin JR 3rd (2024). Two cases of sporadic amyotrophic lateral sclerosis with contrasting clinical phenotypes: genetic insights. Cureus.

[11] Jung YS, Wang W, Jun S (2018). Deregulation of CRAD-controlled cytoskeleton initiates mucinous colorectal cancer via β-catenin. Nat Cell Biol.

[12] Fischer RS, Fritz-Six KL, Fowler VM (2003). Pointed-end capping by tropomodulin3 negatively regulates endothelial cell motility. J Cell Biol.

[13] Disanza A, Carlier MF, Stradal TE (2004). Eps8 controls actin-based motility by capping the barbed ends of actin filaments. Nat Cell Biol.

[14] Chu X, Chen J, Reedy MC, Vera C, Sung KL, Sung LA (2003). E-Tmod capping of actin filaments at the slow-growing end is required to establish mouse embryonic circulation. Am J Physiol Heart Circ Physiol.

[15] Durham JT, Herman IM (2009). Inhibition of angiogenesis in vitro: a central role for beta-actin dependent cytoskeletal remodeling. Microvasc Res.

[16] Padang R, Bagnall RD, Tsoutsman T, Bannon PG, Semsarian C (2015). Comparative transcriptome profiling in human bicuspid aortic valve disease using RNA sequencing. Physiol Genomics.

[17] Wu F, Yuan G, Chen J, Wang C (2017). Network analysis based on TCGA reveals hub genes in colon cancer. Contemp Oncol (Pozn).

[18] Xue Z, Zhu J, Liu J, Wang L, Ding J (2023). Circular RNAs in atrial fibrillation: from bioinformatics analysis of circRNA-miRNA-mRNA network to serum expression. Biochem Biophys Rep.

[19] Peng Y, Song Y, Wang H (2021). Systematic elucidation of the aneuploidy landscape and identification of aneuploidy driver genes in prostate cancer. Front Cell Dev Biol.

[20] Prabhu S, Voskoboinik A, Kaye DM, Kistler PM (2017). Atrial fibrillation and heart failure - cause or effect?. Heart Lung Circ.

[21] Hoshijima M, Chien KR (2002). Mixed signals in heart failure: cancer rules. J Clin Invest.

[22] de Boer RA, Meijers WC, van der Meer P, van Veldhuisen DJ (2019). Cancer and heart disease: associations and relations. Eur J Heart Fail.

[23] Meijers WC, de Boer RA (2019). Common risk factors for heart failure and cancer. Cardiovasc Res.

[24] Kamińska J, Koper-Lenkiewicz OM, Ponikwicka-Tyszko D (2023). New insights on the progesterone (P4) and PGRMC1/NENF complex interactions in colorectal cancer progression. Cancers (Basel).

[25] Zhang Y, Xu Z, Wen W (2022). The microRNA-3622 family at the 8p21 locus exerts oncogenic effects by regulating the p53-downstream gene network in prostate cancer progression. Oncogene.

[26] Gao Y, Liao P (2019). TRPM4 channel and cancer. Cancer Lett.

[27] Kappel S, Stokłosa P, Hauert B (2019). TRPM4 is highly expressed in human colorectal tumor buds and contributes to proliferation, cell cycle, and invasion of colorectal cancer cells. Mol Oncol.

[28] Zhou J, Zhang W, Wei C (2020). Weighted correlation network bioinformatics uncovers a key molecular biosignature driving the left-sided heart failure. BMC Med Genomics.

[29] Wen B, Liu M, Qin X, Mao Z, Chen X (2023). Identifying immune cell infiltration and diagnostic biomarkers in heart failure and osteoarthritis by bioinformatics analysis. Medicine (Baltimore).

[30] Wang GH, Yao L, Xu HW (2013). Identification of MXRA5 as a novel biomarker in colorectal cancer. Oncol Lett.

[31] Morine KJ, Paruchuri V, Qiao X (2016). Endoglin selectively modulates transient receptor potential channel expression in left and right heart failure. Cardiovasc Pathol.

[32] Ghorbel MT, Cherif M, Jenkins E, Mokhtari A, Kenny D, Angelini GD, Caputo M (2010). Transcriptomic analysis of patients with tetralogy of Fallot reveals the effect of chronic hypoxia on myocardial gene expression. J Thorac Cardiovasc Surg.

[33] Tan N, Chung MK, Smith JD (2013). Weighted gene coexpression network analysis of human left atrial tissue identifies gene modules associated with atrial fibrillation. Circ Cardiovasc Genet.

[34] Bernardini M, Brossa A, Chinigo G (2019). Transient receptor potential channel expression signatures in tumor-derived endothelial cells: functional roles in prostate cancer angiogenesis. Cancers (Basel).

[35] Cheng WS, Tao H, Hu EP (2014). Both genes and lncRNAs can be used as biomarkers of prostate cancer by using high throughput sequencing data. Eur Rev Med Pharmacol Sci.

[36] Tharp CA, Haywood ME, Sbaizero O, Taylor MR, Mestroni L (2019). The giant protein titin’s role in cardiomyopathy: genetic, transcriptional, and post-translational modifications of TTN and their contribution to cardiac disease. Front Physiol.

[37] Zheng QX, Wang J, Gu XY, Huang CH, Chen C, Hong M, Chen Z (2021). TTN-AS1 as a potential diagnostic and prognostic biomarker for multiple cancers. Biomed Pharmacother.

